# Association between hypoalbuminemia and mortality in patients undergoing continuous renal replacement therapy: A systematic review and meta-analysis

**DOI:** 10.1371/journal.pone.0283623

**Published:** 2023-03-30

**Authors:** Xuqin Wang, Huan Chu, Huifei Zhou

**Affiliations:** Department of Critical Care Medicine, HuZhou Third Municipal Hospital, Huzhou, Zhejiang, China; Tehran University of Medical Sciences, ISLAMIC REPUBLIC OF IRAN

## Abstract

The review aimed to assess if hypoalbuminemia can predict mortality in patients undergoing continuous renal replacement therapy (CRRT). PubMed, Web of Science, Embase, and CENTRAL were searched for relevant articles published up to 24 July 2022. Adjusted data were pooled to calculate the odds ratio (OR). Sensitivity and meta-regression analysis was conducted. Five studies with 5254 patients were included. Meta-analysis of all five studies demonstrated that hypoalbuminemia was a significant predictor of mortality after CRRT (OR: 1.31 95% CI: 1.07, 1.60 I^2^ = 72% p = 0.01). The results did not change on sensitivity analysis. On meta-regression, we noted that variables like age, male gender, BMI, percentage of diabetics, and pre-CRRT SOFA score had no statistically significant influence on the outcome. Data from a limited number of studies suggest that hypoalbuminemia before initiation of CRRT is an independent predictor of early mortality. Based on current evidence, it may be suggested that patients with low albumin levels initiating CRRT should be prioritized and treated aggressively to reduce adverse outcomes.

## Introduction

Acute kidney injury (AKI) is a major health concern affecting a large population across the world. The disease stems from multiple etiologies and pathophysiological processes wherein there is reduction in kidney function causing retention of waste products, altered electrolyte homeostasis, and variable drug levels. Complete recovery of kidney function is seldom seen with accompanying long-term risk of chronic kidney disease, end-stage renal disease, and death [1, 2]. Data indicates that around 13 million patients are affected by AKI each year and the disease is seen in 60% of patients admitted to the intensive care unit (ICU) [3]. Another systematic review of 213 studies has shown that of all hospitalized patients, one in five adults and one in three children develop AKI [4]. More than a third of AKI patients may develop life threating renal deterioration which requires renal replacement therapy. Amongst the therapeutic modalities, continuous renal replacement therapy (CRRT) is commonly used to manage AKI in the ICU [5]. Patients with AKI have high mortality rates and especially those in need of dialysis the mortality rate reaches up to 50% [6]. Given such high rates of adverse outcome there is a need for accurate and valuable indicators to predict prognosis of patients undergoing CRRT. Also important to note is that the care of such patients involves comprehensive care by physicians as well as nursing personnel. Indeed, nursing personnel are closely involved in the treatment process and should be trained in identifying patients at risk for high mortality.

Albumin is the primary serum protein involved in managing plasma colloid osmotic pressure. It maintains homeostasis between the intracellular fluid, extracellular fluid, and tissue fluid and is also involved in material transport in the blood circulation [7, 8]. Reduced levels of albumin i.e. hypoalbuminemia is commonly seen in hospitalized patients and in a large proportion of critically ill patients [9]. Several studies have shown that hypoalbuminemia is an independent risk factor for increased mortality rates in acute illnesses like septic shock, heart failure, and acute coronary syndrome [10–12]. Research has also shown that hypoalbuminemia is an independent risk factor for development of AKI [13] but there has been limited research if hypoalbuminemia can predict outcomes on patients undergoing CRRT. In the past few years, a number of studies have reported the association between albumin and outcomes of CRRT but with variable results. To the best of our knowledge, no review has been attempted to collate the published evidence. Hence, the current study was designed to pool data from published studies in order to assess if hypoalbuminemia can predict mortality in patients undergoing CRRT.

## Material and methods

### Search strategy

We performed a systematic review and meta-analysis based on the Preferred Reporting Items for Systematic Reviews and Meta-Analyses (PRISMA) recommendations [14]. In conformity with the guidelines, the review was pre-registered on the PROSPERO database (No CRD42022347605). We began by scanning the datasets of PubMed, Web of Science, Embase, and Cochrane Central Register of Controlled Trials (CENTRAL) for relevant articles published up to 24 July 2022. The literature was searched for all types of studies irrespective of the language using the search terms: “albumin”, “hypoalbuminemia”, “continuous renal replacement therapy”, “nutrition”, and “CRRT”. Further details can be found in S1 Table. The search results were consolidated, deduplicated, and screened by title and abstracts by two reviewers separately. Articles of interest to the review were selected and downloaded for full-text analysis. They were cross-checked against the inclusion criteria for final selection. Disagreements between the two reviewers were cleared in consultation with another reviewer. Lastly, we also hand-searched the reference list of included studies and previous reviews to look for any missed articles.

### Eligibility

Inclusion and exclusion criteria are presented in Table 1. While we excluded studies with duplicate data, if two studies were from the same center, the study analyzing the maximum number of patients and reporting relevant outcomes was included. In case of missing data on non-retrievable studies, the authors were contacted by email.

**Table 1:** Inclusion and exclusion criteria.

Inclusion criteria	Exclusion criteria
Studies on patients undergoing CRRT	Studies on peritoneal and hemodialysis
Comparing patients with low and normal albumin levels	Studies not reporting outcomes as adjusted data
Reporting mortality rates as adjusted ratios	Duplicate studies
Using 3 or 3.5g/dl as cut-off for hypoalbuminemia	Review articles, editorials, case reports

### Data management

The following data were extracted from the studies: first author’s name, year of publication, study type and location, sample size, age and gender details, body mass index (BMI), diabetics, AKI, Sequential Organ Failure Assessment (SOFA) score, duration of CRRT, mean albumin levels, the cut-off for hypoalbuminemia, and mortality time.

We assessed the risk of bias using the Newcastle-Ottawa scale (NOS) [15] which has three domains, namely, study population, comparability, and outcomes. Each of them is awarded stars based on predetermined questions. The maximum score achievable is nine.

### Statistical analysis

The meta-analysis was done on “Review Manager” (RevMan, version 5.3; Nordic Cochrane Centre (Cochrane Collaboration), Copenhagen, Denmark; 2014). We extracted adjusted odds ratio (OR) reported by the studies to calculate pooled OR with 95% confidence intervals (CI). The analysis was carried out using a random-effects model. We assessed inter-study heterogeneity using the I^2^ statistic. I^2^ = 25–50% meant low, 50–75% meant medium, and more than 75% meant substantial heterogeneity [16]. Publication bias was assessed by funnel plot and Egger’s test [17]. A sensitivity analysis was performed to examine the influence of each study on the review results. Each study was removed one at a time and the pooled effect estimate was recalculated for the remaining studies. We also explored inter-study heterogeneity using a meta-regression analysis for the variables age, male gender, BMI, diabetics, and SOFA score. The software Meta-Essentials version 1.5 was used for the meta-regression.

## Results

**Fig 1:**
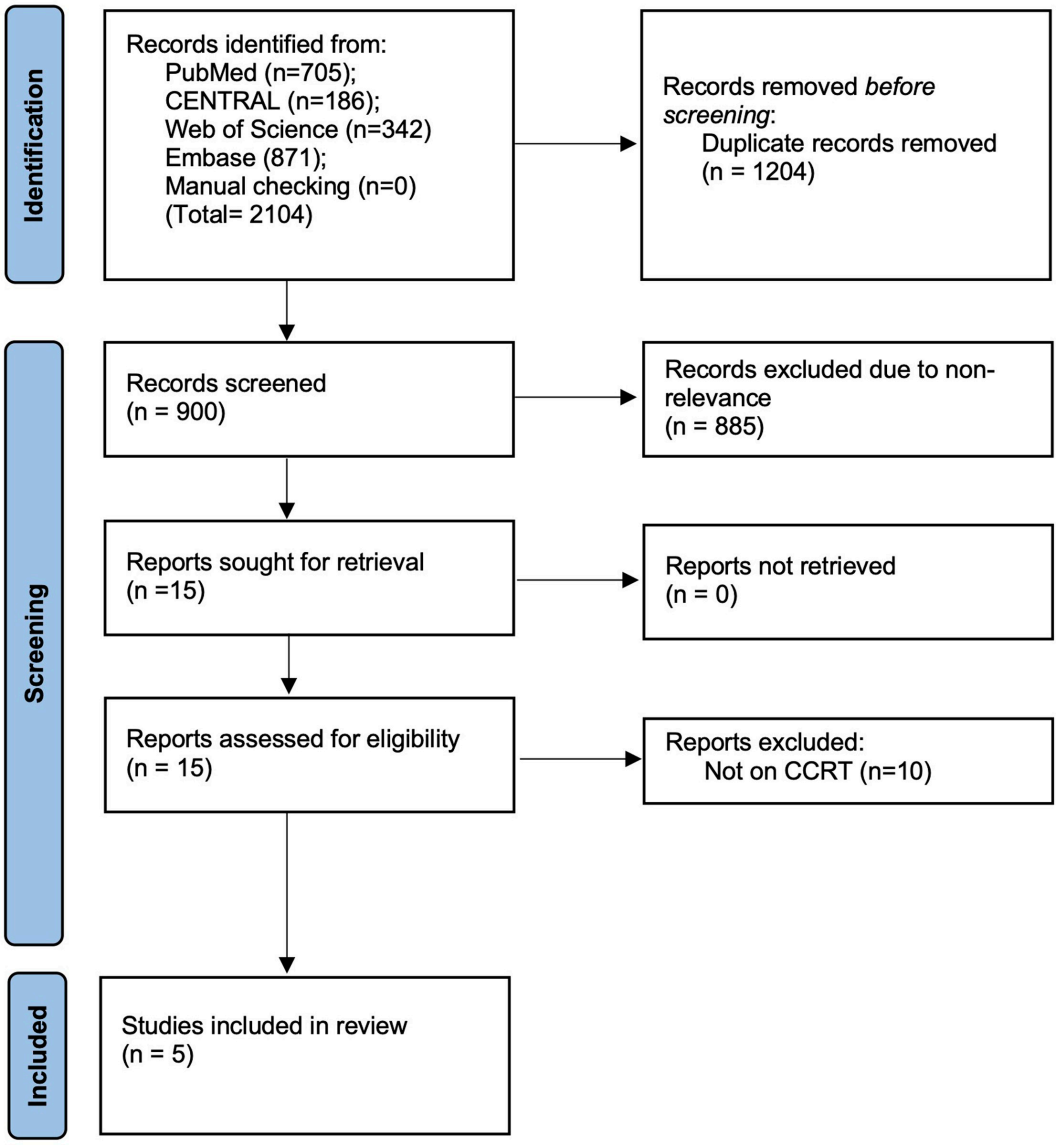
Study flow chart.

Details of the study selection process are presented in Fig 1. The search resulted in 2104 articles. Of these, 900 unique articles were screened and 885 were excluded because of non-relevance. Fifteen articles were downloaded and their full-texts were analyzed. Five fulfilled the inclusion criteria and were included in the review [18–22].

Baseline details extracted from the studies are presented in Table 2. All were retrospective cohort studies published in the past three years. The total sample size of the studies was 5254. The mean age of included patients was more than 59 years. Male gender consisted of 57–63.1% of the total sample of the studies. The mean BMI of the sample was on the higher side (32kg/m^2^) in one study [22]. The percentage of diabetics varied from 57–63.1%. In most studies, the indication of CRRT was only AKI, except for one study wherein AKI patients consisted of 89% of the sample [22]. All studies used 3g/dl as the cut-off for hypoalbuminemia except for one which used 3.5g/dl [22]. All studies examined short-time mortality ranging from in-hospital to 90-day mortality. The NOS score of all studies was 8 (Table 3).

**Table 2:** Details of included studies.

Study	Location	Sample size	Age	Male gender (%)	BMI (kg/m^2^)	Diabetics (%)	AKI (%)	SOFA score before CRRT	Median days of CRRT	Mean albumin level	Cut-off for hypoalbuminemia (g/dl)	Mortality time (days)	Adjusted factors
**Thongprayoon 2022** [22]	USA	911	59.2± 14.9	57	32 ± 15.8	23	89	12.1± 3.7	6 [4–11]	3 ± 0.7	<3.5	90	Age, sex, race, BMI, history of cirrhosis, CCI, CRRT use for AKI, SOFA score, sepsis, mechanical ventilation, vasopressor use, fluid balance from ICU admission to CRRT initiation, ICU type, serum Na, serum K, serum HCO_3_, and arterial pH
**Rhee 2022** [21]	Korea	793	67.5[56–77]	63.1	24.3± 12.9	41.8	100	10.34± 3.74	5[3–8]	3.05± 0.67	<3	In-hospital	Age, sex, BMI, diabetes, liver cirrhosis, COPD, CKD, cancer, sepsis, hemoglobin, SOFA score, number of days from ICU to CRRT, actual delivered CRRT dose, and CRRT duration
**Zheng 2021** [20]	China	837	62.6± 14.4	62.2	23.9± 4.8	31.8	100	12.5± 3.5	NR	NR	<3	90	Age, sex, myocardial infarction, congestive heart failure, cerebrovascular disease, peripheral vascular disease, dementia, diabetes, hypertension, COPD, CRP, GFR, K+, HCO3-, phosphate, BMI, WBC, Hb, BUN, Cr, mechanical ventilation, CCI, APACHE II score, SOFA score, CRRT causes, AKI causes
**Lv 2021** [19]	China	1132	63.3± 14.4	61.7	23.8± 4.6	34.9	100	12.1± 3.5	NR	NR	<3	90	Age; sex, BMI; CCI;CRP; white blood cell; hemoglobin; phosphate; HCO3-; AKI cause; CRRT cause; AKI network stages; 2 h urine output before CRRT initiation; SOFA score
**Moon 2020** [18]	Korea	1581	63.2± 15.2	60.5	NR	29	100	NR	NR	2.7± 0.6	<3	30	Age; sex; weight; acute kidney injury cause; target dose; mean arterial pressure; hypertension; diabetes mellitus; history of ischemic heart disease, cerebrovascular disease, and peripheral vascular disease; malignancy; mechanical ventilation; creatinine, Hb, Na, K levels; and APACHE II score

AKI, acute kidney injury; BMI, body mass index; CCI, Charlson comorbidity index; CRP, C-reactive protein; CRRT, continuous renal replacement therapy; COPD, chronic obstructive pulmonary disease; CKD, chronic kidney disease; NR, not reported; NOS, Newcastle Ottawa scale; SOFA, Sequential Organ Failure Assessment score; GFR, glomerular filtration rate; BUN, blood urea nitrogen; Cr, creatinine; Hb, hemoglobin; WBC, white blood cells

**Table 3:** Risk of bias analysis.

Study	Selection	Comparability	Outcomes	Total score
**Thongprayoon 2022** [22]	****	**	**	8
**Rhee 2022** [21]	****	**	**	8
**Zheng 2021** [20]	****	**	**	8
**Lv 2021** [19]	****	**	**	8
**Moon 2020** [18]	****	**	**	8

**Fig 2:**
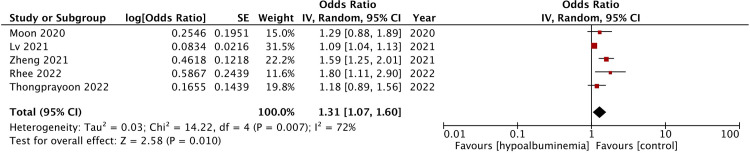
Meta-analysis of the association between hypoalbuminemia and mortality after CRRT.

**Fig 3:**
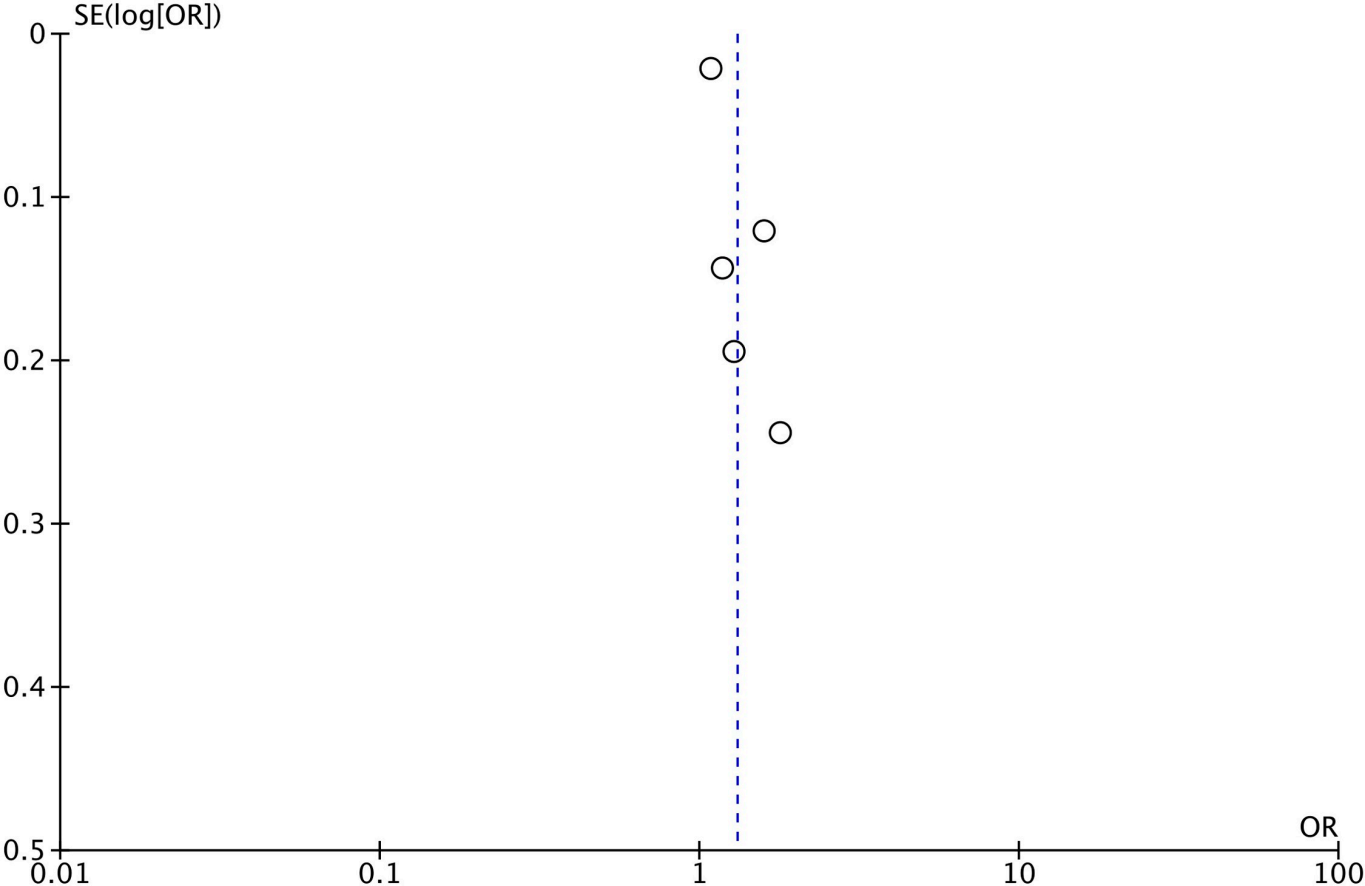
Funnel plot of the meta-analysis of the association between hypoalbuminemia and mortality after CRRT.

Meta-analysis of all five studies demonstrated that hypoalbuminemia was a significant predictor of mortality after CRRT (OR: 1.31 95% CI: 1.07, 1.60 I^2^ = 72% p = 0.01) (Fig 2). There was no gross asymmetry on funnel plot and Egger’s test gave a p-value of 0.775 indicating no publication bias (Fig 3). The results of the sensitivity analysis are shown in Table 4. No study was found to have an undue influence on the study results as the effect size remained statistically significant on the exclusion of any study.

**Table 4:** Results of sensitivity analysis.

Excluded study	Odds ratio
**Thongprayoon 2022** [22]	1.36 95% CI: 1.04, 1.77 I^2^ = 79% p = 0.02
**Rhee 2022** [21]	1.25 95% CI: 1.02, 1.52 I^2^ = 71% p = 0.03
**Zheng 2021** [20]	1.19 95% CI: 1.01, 1.40 I^2^ = 43% p = 0.04
**Lv 2021** [19]	1.42 95% CI: 1.19, 1.69 I^2^ = 18% p = 0.001
**Moon 2020** [18]	1.32 95% CI: 1.04, 1.67 I^2^ = 78% p = 0.02

CI, confidence intervals

Results of the meta-regression analysis are shown in Table 5. We noted that variables like age, male gender, BMI, percentage of diabetics, and pre-CRRT SOFA score had no statistically significant influence on the effect size.

**Table 5:** Meta-regression analysis.

Variable	Beta	SE	-95% CI	+95% CI	P value
**Age**	0.07	0.06	-0.11	0.24	0.29
**Male gender**	0.07	0.07	-0.14	0.28	0.35
**BMI**	-0.02	0.05	-0.19	0.15	0.66
**Diabetics**	0.02	0.03	-0.05	0.10	0.38
**SOFA score**	-0.20	0.22	-0.91	0.50	0.35

BMI, body mass index; CI, confidence intervals; SOFA, Sequential Organ Failure Assessment score; SE, standard error.

## Discussion

The value of validated and accurate prognostic factors for any treatment modality cannot be underestimated. In the case of CRRT, the determination of reliable and easy-to-use prognostic indicators can aid clinicians in timely intervention and allow more focus on patients prone to worse outcomes. In the literature, several studies have explored prognostic indicators for patients undergoing CRRT. Hansrivjit et al. [23] in a meta-analysis have found that age and sepsis were important predictors of early mortality after CRRT. Bai et al. [24] in a recent retrospective study of 846 AKI patients undergoing CRRT have developed a nomogram to predict 28-day mortality. They found that a four-point nomogram consisting of the Charlson comorbidity index, albumin, phosphate, and SOFA score can effectively predict mortality after CRRT.

Serum albumin levels are easy to measure and hence have been used to predict mortality in several conditions. Wang et al. [25] have demonstrated that albumin levels are predictive of mortality in patients undergoing peritoneal dialysis. In another study, Leite et al. [26] found that low albumin levels before ICU admission were predictive of early mortality, prolonged duration of mechanical ventilation, and lower probability of ICU discharge in critically ill pediatric patients. Similarly, Atrash et al. [27] have noted a significant relationship between hypoalbuminemia and early mortality in adult critically ill patients. Nilzeki et al. [28] have shown that low albumin level is a strong and independent predictor of adverse prognosis in patients with chronic heart failure. Another study by Akerblom et al. [29] have noted a significant relationship between hypoalbuminemia and increased morbidity and mortality in acute coronary syndrome patients independent of glomerular filtration rate. Mok et al. [30] found albumin levels to be an independent predictor of poor prognosis in myocardial infarction patients.

In our review, we focused on examining the relationship between low albumin levels and early mortality after CRRT. On meta-analysis of data from 5254 patients, we noted that hypoalbuminemia before initiation of CRRT was a significant predictor of early mortality. The pooled OR was 1.31 indicating a 31% increased risk of mortality after CRRT. Important to note was that the results did not vary on sensitivity analysis and no study had an undue effect on the overall outcome. Furthermore, the funnel plot also failed to demonstrate any publication bias. Nevertheless, it is important to note that the overall heterogeneity in the analysis was high at 72%. While a myriad of confounding variables can influence mortality rates in critically ill patients undergoing CRRT, we could analyze only a limited number of factors in the meta-regression analysis. The scarcity of reporting of common baseline variables like duration of CRRT, mean baseline albumin levels, etc. along with the limited number of studies in literature precluded a more comprehensive meta-regression. The limited data failed to show a relationship between age, male gender, BMI, percentage of diabetics, pre-CRRT SOFA score and the overall outcome.

The relationship between albumin levels and early mortality rates after CRRT can be explained via several pathophysiological processes. Foremost, albumin is the major serum protein that maintains capillary membrane permeability by regulating colloid oncotic pressure and intravascular volume. In conditions leading to hypoalbuminemia, the capillary permeability is increased which causes a fluid shift from intravascular to extravascular compartment causing edema and reduced mean arterial pressure [7, 8]. Recent research has shown that both fluid overload and lower blood pressure are independent predictors of mortality in patients undergoing CRRT [31, 32]. Also, albumin is an indicator of the inflammatory and nutritional state of an individual. The presence of hypoalbuminemia is associated with a pro-inflammatory state with the production of endotoxins, chemokines, and cytokines. These inflammatory mediators lead to decreased synthesis of albumin and increased albumin leakage which in turn promotes fluid overload [33]. Albumin is also the primary transporter of drugs and metabolites and hence hypoalbuminemia may influence the pharmacokinetics of drugs used to treat severely ill patients thereby reducing its efficacy [34]. Lastly, the antioxidant property of albumin helps in scavenging free oxygen which reduced free radical production and oxidative injury. Reduced albumin levels may increase the risk of oxidative injury and apoptosis [35].

The strength of the study is that it is the first meta-analysis to assess the relationship between hypoalbuminemia and outcomes after CRRT. All of the included studies were recently published thereby providing contemporary evidence. We also analyzed only adjusted outcomes from the studies to negate the role of confounding variables. Appropriate sensitivity analysis and meta-regression were performed to explore the source of heterogeneity in the meta-analysis.

Nevertheless, there are limitations to the review. Firstly, only five studies were available for analysis. The limited number of studies precluded a detailed meta-regression and further subgroup analyses. Also, the retrospective nature of the studies could have introduced bias in the results. Secondly, as mentioned earlier, the relationship between albumin and mortality can be confounded by several variables. We tried to negate this by including only multivariable-adjusted data from the studies but other unknown confounders could have been missed. Also, the adjusted confounders were not the same in included studies, which is indeed a source of bias. One important confounder is the status of albumin transfusion during CRRT which was not available in the majority of studies. Albumin transfusions can alter albumin levels and it is currently unknown how it may impact mortality. Lastly, the majority of the included studies were on Asian patients with only one study on the Western population. This may affect the generalizability of the results.

## Conclusions

Data from a limited number of studies suggest that hypoalbuminemia before initiation of CRRT is an independent predictor of early mortality. Based on current evidence, it may be suggested that physicians as well as nursing personnel should identify patients with low albumin levels and the treatment of such individuals should be prioritized to reduce adverse outcomes. Also, further prospective studies should be conducted to supplement current evidence on the relationship between albumin and mortality after CRRT.

## Supporting information

S1 Table Search strategy. (DOCX)

S1 File PRISMA checklist. (DOCX)
