# Supplementary material for: Association between hypoalbuminemia and mortality in patients undergoing continuous renal replacement therapy: A systematic review and meta-analysis
Source: PLoS One. 2023 Mar 30;18(3):e0283623. doi: 10.1371/journal.pone.0283623 (PMC10062548; doi:10.1371/journal.pone.0283623)
Supplement: S1 Table — (DOCX) [file pone.0283623.s001.docx]

**S1 Table. Search strategy.**

**PUBMED**

| **Query** | **Sort By** | **Filters** | **Search Details** |
| --- | --- | --- | --- |
| ((albumin) OR (hypoalbuminemia)) AND (continuous renal replacement therapy) | Most Recent |  | ("albumin s"[All Fields] OR "albumine"[All Fields] OR "albumines"[All Fields] OR "albumins"[MeSH Terms] OR "albumins"[All Fields] OR "albumin"[All Fields] OR ("hypoalbuminaemia"[All Fields] OR "hypoalbuminemia"[MeSH Terms] OR "hypoalbuminemia"[All Fields])) AND ("continuous renal replacement therapy"[MeSH Terms] OR ("continuous"[All Fields] AND "renal"[All Fields] AND "replacement"[All Fields] AND "therapy"[All Fields]) OR "continuous renal replacement therapy"[All Fields]) |
| (nutrition) AND (continuous renal replacement therapy) | Most Recent |  | ("nutrition s"[All Fields] OR "nutritional status"[MeSH Terms] OR ("nutritional"[All Fields] AND "status"[All Fields]) OR "nutritional status"[All Fields] OR "nutrition"[All Fields] OR "nutritional sciences"[MeSH Terms] OR ("nutritional"[All Fields] AND "sciences"[All Fields]) OR "nutritional sciences"[All Fields] OR "nutritional"[All Fields] OR "nutritionals"[All Fields] OR "nutritions"[All Fields] OR "nutritive"[All Fields]) AND ("continuous renal replacement therapy"[MeSH Terms] OR ("continuous"[All Fields] AND "renal"[All Fields] AND "replacement"[All Fields] AND "therapy"[All Fields]) OR "continuous renal replacement therapy"[All Fields]) |
| ((albumin) OR (hypoalbuminemia)) AND (CRRT) | Most Recent |  | ("albumin s"[All Fields] OR "albumine"[All Fields] OR "albumines"[All Fields] OR "albumins"[MeSH Terms] OR "albumins"[All Fields] OR "albumin"[All Fields] OR ("hypoalbuminaemia"[All Fields] OR "hypoalbuminemia"[MeSH Terms] OR "hypoalbuminemia"[All Fields])) AND "CRRT"[All Fields] |
| (nutrition) AND (CRRT) | Most Recent |  | ("nutrition s"[All Fields] OR "nutritional status"[MeSH Terms] OR ("nutritional"[All Fields] AND "status"[All Fields]) OR "nutritional status"[All Fields] OR "nutrition"[All Fields] OR "nutritional sciences"[MeSH Terms] OR ("nutritional"[All Fields] AND "sciences"[All Fields]) OR "nutritional sciences"[All Fields] OR "nutritional"[All Fields] OR "nutritionals"[All Fields] OR "nutritions"[All Fields] OR "nutritive"[All Fields]) AND "CRRT"[All Fields] |

**EMBASE**

#1. 'albumin'/exp OR 'albumin'

#2. 'hypoalbuminemia'

#3. #1 OR #2

#4. ‘nutrition’

#5. 'continuous renal replacement therapy'

#6. #3 AND #5

#7. #4 AND #5

**CENTRAL**

#1 ((albumin) OR (hypoalbuminemia)) AND (continuous renal replacement therapy)

#2 (nutrition) AND (continuous renal replacement therapy)

**WEB OF SCIENCE**

#1 ((albumin) OR (hypoalbuminemia)) AND (continuous renal replacement therapy)

#2 (nutrition) AND (continuous renal replacement therapy)
